# Estimated changes in free sugar consumption one year after the UK soft drinks industry levy came into force: controlled interrupted time series analysis of the National Diet and Nutrition Survey (2011–2019)

**DOI:** 10.1136/jech-2023-221051

**Published:** 2024-07-09

**Authors:** Nina Trivedy Rogers, Steven Cummins, Catrin P Jones, Oliver Mytton, Mike Rayner, Harry Rutter, Martin White, Jean Adams

**Affiliations:** 1 MRC Epidemiology Unit, University of Cambridge School of Clinical Medicine, Institute of Metabolic Science, Cambridge Biomedical Campus, University of Cambridge, Cambridge, UK; 2 Department of Public Health, Environments & Society, London School of Hygiene & Tropical Medicine, London, UK; 3 Great Ormond Street Institute of Child Health, University College London, London, UK; 4 Nuffield Department of Population Health, University of Oxford, Oxford, UK; 5 Department of Social and Policy Sciences, , University of Bath, Bath, UK

**Keywords:** NUTRITION, PUBLIC HEALTH, DIET

## Abstract

**Background:**

The UK soft drinks industry levy (SDIL) was announced in March 2016 and implemented in April 2018, encouraging manufacturers to reduce the sugar content of soft drinks. This is the first study to investigate changes in individual-level consumption of free sugars in relation to the SDIL.

**Methods:**

We used controlled interrupted time series (2011–2019) to explore changes in the consumption of free sugars in the whole diet and from soft drinks alone 11 months after SDIL implementation in a nationally representative sample of adults (>18 years; n=7999) and children (1.5–19 years; n=7656) drawn from the UK National Diet and Nutrition Survey. Estimates were based on differences between observed data and a counterfactual scenario of no SDIL announcement/implementation. Models included protein consumption (control) and accounted for autocorrelation.

**Results:**

Accounting for trends prior to the SDIL announcement, there were absolute reductions in the daily consumption of free sugars from the whole diet in children and adults of 4.8 g (95% CI 0.6 to 9.1) and 10.9 g (95% CI 7.8 to 13.9), respectively. Comparable reductions in free sugar consumption from drinks alone were 3.0 g (95% CI 0.1 to 5.8) and 5.2 g (95% CI 4.2 to 6.1). The percentage of total dietary energy from free sugars declined over the study period but was not significantly different from the counterfactual.

**Conclusion:**

The SDIL led to significant reductions in dietary free sugar consumption in children and adults. Energy from free sugar as a percentage of total energy did not change relative to the counterfactual, which could be due to simultaneous reductions in total energy intake associated with reductions in dietary free sugar.

**WHAT IS ALREADY KNOWN ON THIS TOPIC**

- High intakes of free sugars are associated with a range of non-communicable diseases. Sugar sweetened beverages constitute a major source of dietary free sugars in children and adults.
- The UK soft drink industry levy (SDIL) led to a reduction in the sugar content in many sugar sweetened beverages and a reduction in household purchasing of sugar from drinks.
- No previous study has examined the impact of the SDIL on total dietary consumption of free sugars at the individual level.

**WHAT THIS STUDY ADDS**

- There were declining trends in the intake of dietary free sugar in adults and children prior to the UK SDIL.
- Accounting for prior trends, 1 year after the UK SDIL came into force children and adults further reduced their free sugar intake from food and drink by approximately 5 g/day and 11 g/day, respectively. Children and adults reduced their daily free sugar intake from soft drinks alone by approximately 3 g/day and approximately 5 g/day, respectively.
- Energy intake from free sugars as a proportion of total energy consumed did not change significantly following the UK SDIL, indicating energy intake from free sugar was reducing simultaneously with overall total energy intake.

**HOW THIS STUDY MIGHT AFFECT RESEARCH, PRACTICE OR POLICY**

- The UK SDIL was associated with significant reductions in consumption of free sugars from soft drinks and across the whole diet and reinforces previous research indicating a reduction in purchasing. This evidence should be used to inform policy when extending or considering other sugar reduction strategies.
- Energy intake from free sugars has been falling but levels remain higher than the 5% recommendation set by the WHO. Reductions in dietary sugar in relation to the SDIL may have driven significant reductions in overall energy.

## Introduction

High consumption of free sugars is associated with non-communicable diseases.^1^ Guidelines from the World Health Organization (WHO) and the UK Scientific Advisory Committee on Nutrition (SACN) suggest limiting free sugar consumption to below 5% of total energy intake to achieve maximum health benefits,^1 2^ equivalent to daily maximum amounts of 30 g for adults, 24 g for children (7–10 years) and 19 g for young children (4–6 years). In the UK, consumption of free sugar is well above the recommended daily maximum, although levels have fallen over the last decade.^3^ For example, adolescents consume approximately 70 g/day^4^ and obtain 12.3% of their energy from free sugars.^3^ Sugar sweetened beverages (SSBs) constitute a major source of free sugar in the UK diet,^2 5^ and are the largest single source for children aged 11–18 years where they make up approximately one-third of their daily sugar intake.^6^ A growing body of evidence has shown a link between consumption of SSBs and higher risk of weight gain, type 2 diabetes, coronary heart disease and premature mortality,^7^ such that the WHO recommends taxation of SSBs in order to reduce over-consumption of free sugars and to improve health.^8^ To date, >50 countries have introduced taxation on SSBs, which has been associated with a reduction in sales and dietary intake of free sugar from SSBs.^9^ Reductions in the prevalence of childhood obesity^10 11^ and improvements in dental health outcomes^12 13^ have also been reported.

In March 2016 the UK government announced the UK soft drink industry levy (SDIL), a two-tier levy on manufacturers, importers and bottlers of soft drinks which would come into force in March 2018.^14^ The levy was designed to incentivise manufacturers to reformulate and reduce the free sugar content of SSBs (see details in online supplemental text 1).

10.1136/jech-2023-221051.supp1 Supplementary data

One year after the UK SDIL was implemented there was evidence of a reduction in the sugar content of soft drinks^15^ and households on average reduced the amount of sugar purchased from soft drinks by 8 g/week with no evidence of substitution with confectionary or alcohol.^16^ However, lack of available data meant it was not possible to examine substitution of purchasing other sugary foods and drinks, which has previously been suggested in some but not all studies.^17 18^ Household purchasing only approximates individual consumption because it captures only those products brought into the home, products may be shared unequally between household members, and it does not account for waste.

To examine the effects of the SDIL on total sugar intake at the individual level, in this study we used surveillance data collected using 3- or 4-day food diaries as part of the UK National Diet and Nutrition Survey (NDNS). We aimed to examine changes in absolute and relative consumption of free sugars from soft drinks alone and from both food and drinks (allowing us to consider possible substitutions with other sugary food items), following the announcement and implementation of the UK SDIL.

## Methods

### Data source

We used 11 years of data (2008–2019) from the NDNS. Data collection, sampling design and information on response is described in full elsewhere.^19^ In brief, NDNS is a continuous national cross-sectional survey capturing information on food consumption, nutritional status and nutrient intake inside and outside of the home in a representative annual sample of approximately 500 adults and 500 children (1.5–18 years) living in private households in the UK. Participants are sampled throughout the year, such that in a typical month about 40 adults and 40 children participate (further details are shown in online supplemental text 2).

### Outcomes of interest

Outcomes of interest were absolute and relative changes in the total intake of dietary free sugar from (1) all food and soft drinks combined and (2) from soft drinks alone. A definition of free sugar is given in online supplemental text 3. Drink categories examined were those that fell within the following NDNS categories: soft drinks – not low calorie; soft drinks – low calorie; semi-skimmed milk; whole milk; skimmed milk; fruit juice, 1% fat milk and other milk and cream. Additionally, we examined absolute and relative changes in percentage energy from free sugar in (1) food and soft drinks and (2) soft drinks alone. While examination of changes in sugar consumption and percentage energy from sugar across the whole diet (food and drink) captures overall substitutions with other sugar-containing products following the UK SDIL, examination of sugar consumption from soft drinks alone provides a higher level of specificity to the SDIL.

Protein intake was selected as a non-equivalent dependent control. It was not a nutritional component specifically targeted by the intervention or other government interventions and therefore is unlikely to be affected by the SDIL but could still be affected by confounding factors such as increases in food prices^20^ (see online supplemental text 4).

### Statistical analysis

Controlled interrupted time series (ITS) analyses were performed to examine changes in the outcomes in relation to the UK SDIL separately in adults and children. We analysed data at the quarterly level over 11 years with the first data point representing dates from April to June 2008 and the last representing dates from January to March 2019. Model specifications are shown in online supplemental text 5. Where diary date entries extended over two quarters, the earlier quarter was designated as the time point for analysis. Generalised least squares models were used. Autocorrelation in the time series was determined using Durbin–Watson tests and from visualisations of autocorrelation and partial correlation plots. Autocorrelation-moving average correlation structure with order (p) and moving average (q) parameters were used and selected to minimise the Akaike information criterion in each model. Trends in free sugar consumption prior to the announcement of SDIL in April 2016 were used to estimate counterfactual scenarios of what would have happened if the SDIL had not been announced or come into force. Thus, the interruption point was the 3-month period beginning April 2016. Absolute and relative differences in consumption of free sugars/person/day were estimated by calculating the difference between the observed and counterfactual values at quarterly time point 45. To account for non-response and to ensure the sample distribution represented the UK distribution of females and males and age profile, weights provided by NDNS were used and adapted for analysis of adults and children separately.^21^A study protocol has been published^22^ and the study is registered (ISRCTN18042742). For changes to the original protocol see online supplemental text 6. All statistical analyses were performed in R version 4.1.0.

## Results

Data from 7999 adults and 7656 children were included across 11 years representing approximately 40 children and 40 adults each month. Table 1 gives descriptive values for the outcomes of interest. Compared with the pre-announcement period, free sugars consumed from all soft drinks reduced by around one-half in children and one-third in adults in the post-announcement period. Total dietary free sugar consumption and percentage of total dietary energy derived from free sugars also declined. Mean protein consumption was relatively stable over both periods in children and adults. The age and sex of the children and adults were very similar in the pre- and post-announcement periods.

**Table 1:** Mean amount of free sugar (g) consumed in children and adults per day during the study period before and after the announcement of the soft drinks industry levy (SDIL)

	Children		Adults	
	Pre-announcement*	Post-announcement†	Pre-announcement*	Post-announcement†
Age (years)	9.5 (5.2)	9.5 (5.2)	52.7 (19.8)	51.3 (18.7)
Sex (female), N (%)	2908 (48.9)	841 (49.0)	3618 (58.6)	1081 (58.8)
Free sugar (g/day)				
Free sugar from soft drinks only	22.0 (4.4)	12.0 (2.2)	15.3 (3.1)	10.0 (2.6)
Free sugar from food and soft drinks	62.4 (6.0)	47.8 (3.6)	57.9 (3.6)	47.9 (3.3)
Energy (from free sugar/protein) (%)				
Energy from free sugar in soft drinks as % of energy in soft drinks	48.1 (12.3)	26.3 (2.8)	34.3 (2.3)	22.8 (2.3)
Energy from free sugar in food and soft drinks as % of total dietary energy	16.7 (4.1)	9.9 (1.2)	12.7 (2.4)	8.8 (0.8)
Energy from protein in soft drinks as % of total energy in soft drinks	15.8 (2.6)	14.7 (1.1)	21.2 (4.0)	18.4 (1.2)
Energy from protein in food and soft drinks as % of total energy	16.7 (3.3)	12.4 (0.9)	18.0 (3.3)	14.8 (1.0)
Protein (g/day)				
Protein from soft drinks only	6.6 (0.6)	6.3 (0.6)	5.7 (0.5)	5.4 (0.4)
Protein from food and soft drinks	58.0 (2.0)	56.2 (1.5)	74.1 (2.6)	73.8 (2.2)

*April 2008 to March 2016.

†April 2016 to January 2019.

All estimates of change in free sugar consumption referred to below are based on g/individual/day in the 3-month period beginning January 2019 and compared with the counterfactual scenario of no UK SDIL announcement and implementation.

### Change in free sugar consumption (soft drinks only)

**Figure 1:**
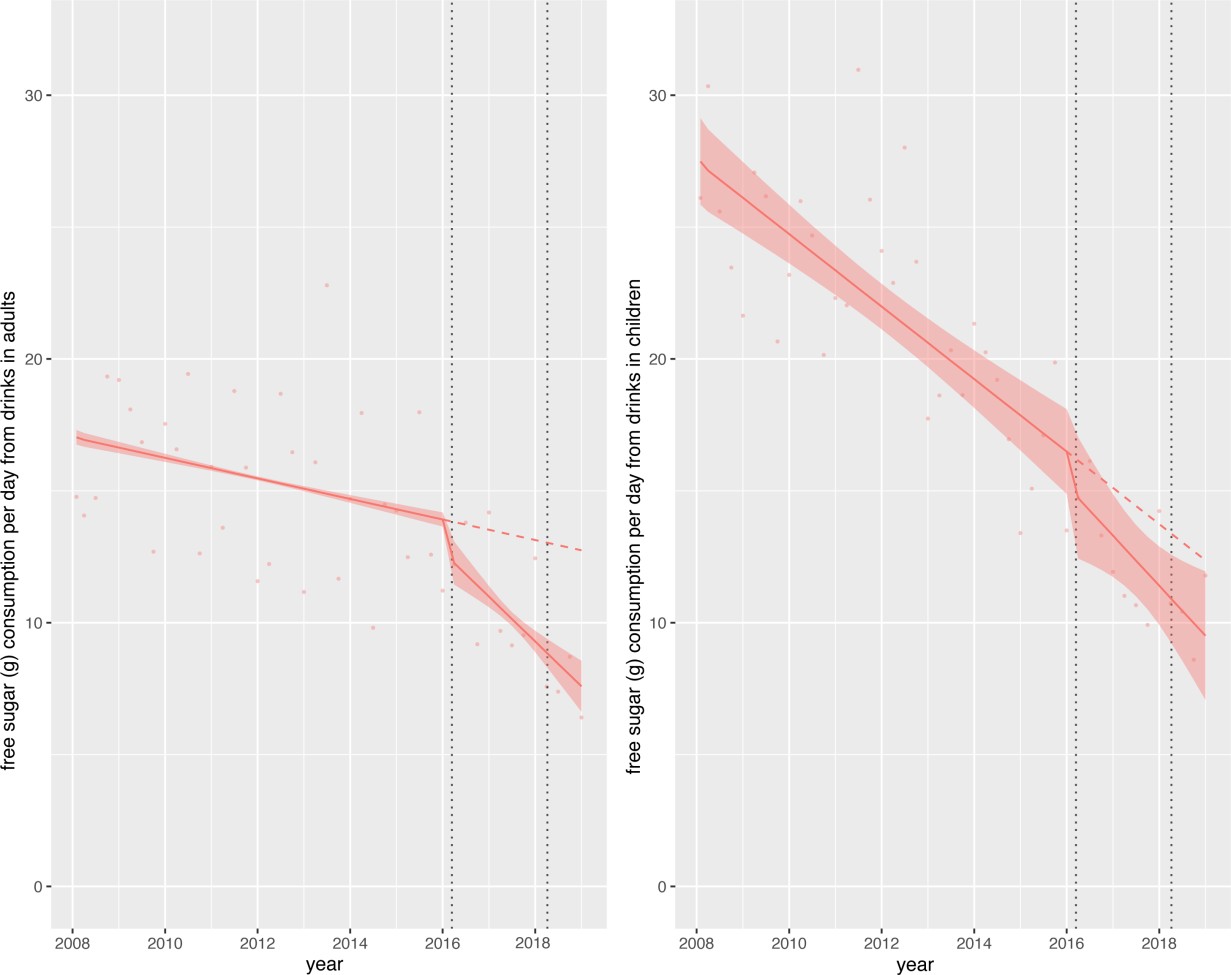
Observed and modelled daily consumption (g) of free sugar from drink products per adult/child from April 2008 to March 2019. Red points show observed data and solid red lines (with light red shadows) show modelled data (and 95% CIs) of free sugar consumed from drinks. The dashed red line indicates the counterfactual line based on pre-announcement trends and if the announcement and implementation had not happened. Modelled protein consumption from drinks (control group) was removed from the graph to include resolution but is available in the supplementary section. The first and second dashed lines indicate the announcement and implementation of the soft drinks industry levy (SDIL), respectively.

In children, consumption of free sugars from soft drinks was approximately 27 g/day at the start of the study period but fell steeply throughout. By the end of the study period mean sugar consumption from soft drinks was approximately 10 g/day (figure 1). Overall, relative to the counterfactual scenario, there was an absolute reduction in daily free sugar consumption from soft drinks of 3.0 g (95% CI 0.1 to 5.8) or a relative reduction of 23.5% (95% CI 46.0% to 0.9%) in children (table 2). In adults, free sugar consumption at the beginning of the study was lower than that of children (approximately 17 g/day) and was declining prior to the SDIL announcement, although less steeply (figure 1). Following the SDIL announcement, free sugar consumption from soft drinks appeared to decline even more steeply. There was an absolute reduction in free sugar consumption from soft drinks of 5.2 g (95% CI 4.2 to 6.1) or a relative reduction of 40.4% (95% CI 32.9% to 48.0%) in adults relative to the counterfactual (figure 1, table 2).

**Table 2:** Change in free sugar consumption in food and drink and energy from free sugar as a proportion of total energy compared with the counterfactual scenario of no announcement and implementation of the UK soft drinks industry levy (SDIL)

	Children		Adults	
	Absolute change (g)	Relative change (%)	Absolute change (g)	Relative change (%)
Free sugar from soft drinks only	**−3.0 (−5.8, −0.1**)	**−23.5 (−46.0, −0.9**)	**−5.2 (−6.1, −4.2**)	**−40.4 (−48.0, −32.9**)
Free sugar from food and soft drinks	**−4.8 (−9.1, −0.6**)	**−9.7 (−18.2, −1.2**)	**−10.9 (−13.9, −7.8**)	**−19.8 (−25.4, −14.2**)
Energy from free sugar in food and soft drinks as % of total energy (%)	−0.7 (−3.9, 2.5)	−7.6 (−41.7, 26.5)	−2.6 (0.6, −5.8)	−24.3 (−54.0, 5.4)
Energy from free sugar in soft drinks as % of total energy in soft drinks (%)	0.4 (−7.1, 8.0)	1.8 (−30.7, 34.3)	−0.52 (−5.4, 4.3)	−2.4 (−24.6, 19.8)

### Change in total dietary free sugar consumption (food and soft drinks combined)

**Figure 2:**
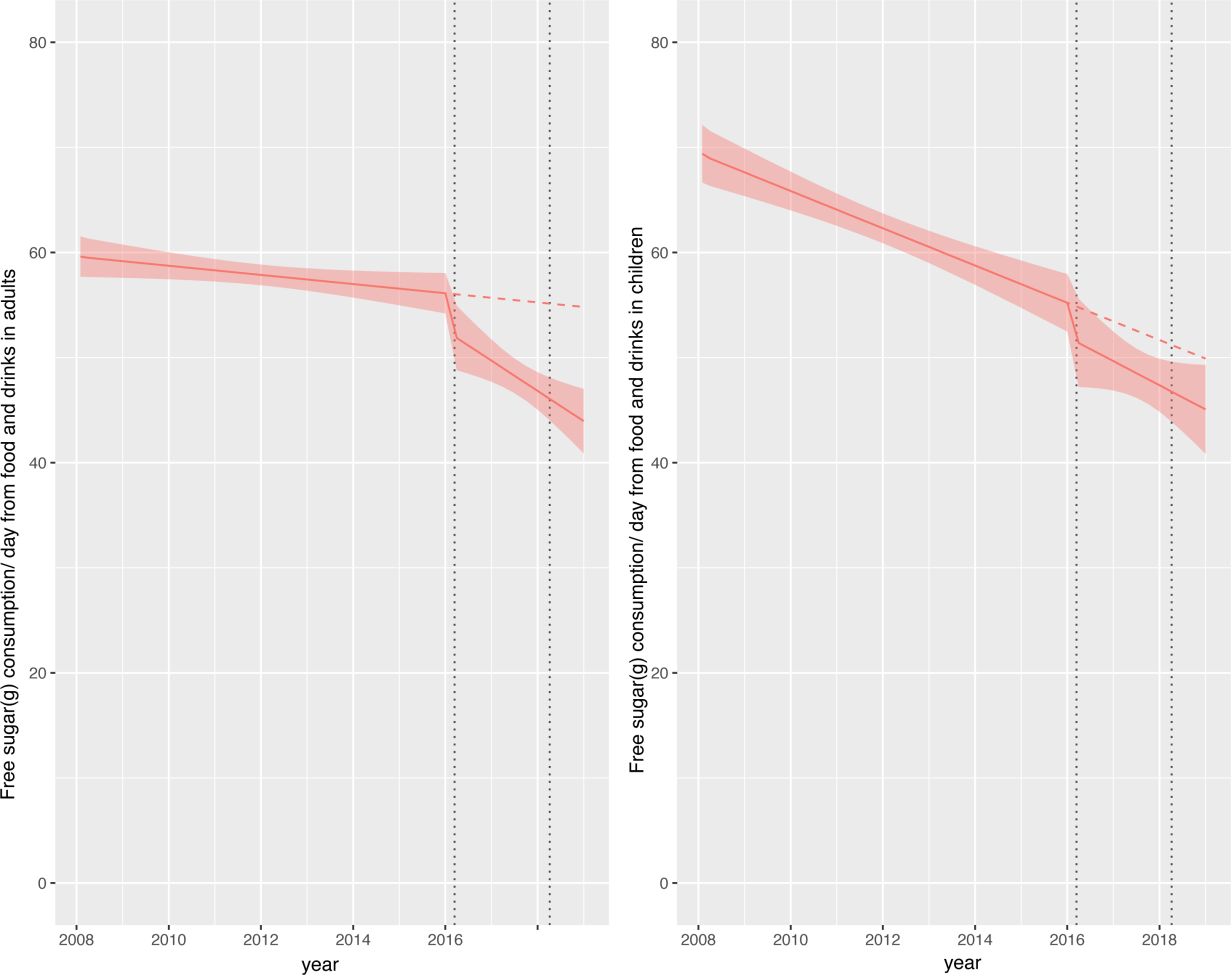
Observed and modelled daily consumption (g) of free sugar from food and drink products per adult/child from April 2008 to March 2019. Red points show observed data and solid red lines (with light red shadows) show modelled data (and 95% CIs) of free sugar consumed from food and drinks. The dashed red line indicates the counterfactual line based on pre-announcement trends and if the announcement and implementation had not happened. Modelled protein consumption from food and drinks (control group) was removed from the graph to include resolution but is available in the supplementary section. The first and second dashed lines indicate the announcement and implementation of the soft drinks industry levy (SDIL), respectively.

Consumption of total dietary free sugars in children was approximately 70 g/day at the beginning of the study but this fell to approximately 45 g/day by the end of the study (figure 2). Relative to the counterfactual scenario, there was an absolute reduction in total dietary free sugar consumption of 4.8 g (95% CI 0.6 to 9.1) or relative reduction of 9.7% (95% CI 18.2% to 1.2%) in children (figure 2; table 2). In adults, consumption of total dietary free sugar consumption at the beginning of the study was approximately 60 g/day falling to approximately 45 g/day by the end of the study (figure 2). Relative to the counterfactual scenario there was an absolute reduction in total dietary free sugar consumption in adults of 10.9 g (95% CI 7.8 to 13.9) or a relative reduction of 19.8% (95% CI 25.4% to 14.2%). Online supplemental figures show that, relative to the counterfactual, dietary protein consumption and energy from protein was more or less stable across the study period (see online supplemental figures S3–S6).

### Change in energy from free sugar as a proportion of total energy

The percentage of energy from total dietary free sugar decreased across the study period but did not change significantly relative to the counterfactual scenario in children or adults, with relative changes in free sugar consumption of −7.6 g (95% CI −41.7 to 26.5) and −24.3 g (95% CI −54.0 to 5.4), respectively (see online supplemental figure S1 and table 2). Energy from free sugar in soft drinks as a proportion of total energy from soft drinks also decreased across the study period but did not change significantly relative to the counterfactual (see online supplemental figure S2).

## Discussion

### Summary of main findings

This study is the first to examine individual level consumption of free sugars in the total diet (and in soft drinks only) in relation to the UK SDIL. Using nationally representative population samples, we found that approximately 1 year following the UK SDIL came into force there was a reduction in total dietary free sugar consumed by children and adults compared with what would have been expected if the SDIL had not been announced and implemented. In children this was equivalent to a reduction of 4.8 g of free sugars/day from food and soft drinks, of which 3 g/day came from soft drinks alone, suggesting that the reduction of sugar in the diet was primarily due to a reduction of sugar from soft drinks. In adults, reductions in dietary sugar appeared to come equally from food and drink with an 11 g reduction in food and drink combined, of which 5.2 g was from soft drinks only. There was no significant reduction compared with the counterfactual in the percentage of energy intake from free sugars in the total diet or from soft drinks alone in both children and adults, suggesting that energy intake from free sugar was reducing simultaneously with overall total energy intake.

### Comparison with other studies and interpretation of results

Our finding of a reduction in consumption of free sugars from soft drinks after accounting for pre-SDIL announcement trends is supported by previous research showing a large reduction in the proportion of available soft drinks with over 5 g of sugar/100 mL, the threshold at which soft drinks become levy liable.^15^ Furthermore, efforts of the soft drink industry to reformulate soft drinks were found to have led to significant reductions in the volume and per capita sales of sugar from these soft drinks.^23^

Our findings are consistent with recent research showing reductions in purchasing of sugar from soft drinks of approximately 8 g/household/week (equivalent to approximately 3 g/person/week or approximately 0.5 g/person/day) 1 year after the SDIL came into force.^16^ The estimates from the current study suggest larger reductions in consumption (eg, 3 g free sugar/day from soft drinks in children) than previously reported for purchasing. Methodological differences may explain these differences in estimated effect sizes. Most importantly, the previous study used data on soft drink purchases that were for consumption in the home only. In contrast, we captured information on consumption (rather than purchasing) in and out of the home. Consumption of food and particularly soft drinks outside of the home in young people (1–21 years) increases with age and makes a substantial contribution to total free sugar intakes, highlighting the importance of recording both in home and out of home sugar consumption.^4^ Purchasing and consumption data also treat waste differently; purchase data record what comes into the home and therefore include waste, whereas consumption data specifically aim to capture leftovers and waste and exclude it from consumption estimates. While both studies use weights to make the population samples representative of the UK, there may be differences in the study participant characteristics in the two studies, which may contribute to the different estimates.

Consistent with other studies,^24^ we found that across the 11-year study period we observed a downward trend in free sugar and energy intake in adults and children.^3^ A decline in consumption of free sugars was observed in the whole diet rather than just soft drinks, suggesting that consumption of free sugar from food was also declining from as early as 2008. One reason might be the steady transition from sugar in the diet to low-calorie artificial sweeteners, which globally have had an annual growth of approximately 5.1% between 2008 and 2015.^25^

Public health signalling around the time of the announcement of the levy may also have contributed to the changes we observed. Public acceptability and perceived effectiveness of the SDIL was reported to be high 4 months before and approximately 20 months after the levy came into force.^26^ Furthermore, awareness of the SDIL was found to be high among parents of children living in the UK, with most supporting the levy and intending to reduce purchases of SSBs as a result.^27^ Health signalling was also found following the implementation of the SSB tax in Mexico, with one study reporting that most adults (65%) were aware of the tax and that those aware of the tax were more likely to think the tax would reduce purchases of SSBs,^28^ although a separate study found that adolescents in Mexico were mostly unaware of the tax,^29^ suggesting that public health signalling may differ according to age.

In 2016 the UK government announced a voluntary sugar reduction programme as part of its childhood obesity plan (which also included SDIL) with the aim of reducing sugar sold by industry by 5% no later than 2018 and by 20% in time for 2020 through both reformulation and portion size reduction.^30^ While the programme only managed to achieve overall sugar reductions of approximately 3.5%, this did include higher reductions in specific products such as yoghurts (−17%) and cereals (−13%) by 2018 which may have contributed to some of the observed reductions in total sugar consumption (particularly from foods) around the time of the SDIL. While there is strong evidence that the UK SDIL led to significant reformulation^15^ and reductions in purchases of sugar from soft drinks,^16^ the products targeted by the sugar reduction programme were voluntary with no taxes or penalties if targets were not met, possibly leading to less incentive for manufacturers to reformulate products that were high in sugar. The 5-year duration of the voluntary sugar reduction programme also makes it challenging to attribute overall reductions using interruption points that we assigned to the ITS to align with the date of the SDIL announcement. The soft drinks categories in our study included levy liable and non-levy liable drinks because we wanted to examine whether individuals were likely to substitute levy liable drinks for high sugar non-liable options. The decline in sugar consumed overall and in soft drinks in relation to the levy suggests that individuals did not change their diets substantially by substituting more sugary foods and drinks. This is consistent with findings from a previous study that found no changes in relation to the levy in sugar purchased from fruit juice, powder used to make drinks or confectionery.^16^

Consistent with previous analyses,^3^ our findings showed that there was a downward trend in energy intake from sugar as a proportion of total energy across the duration of the study. While there was no reduction compared with the counterfactual scenario (which was also decreasing), our estimates suggest that, by 2019, on average energy from sugar as a proportion of all energy appears to be in line with the WHO recommendation of 10% but not the more recent guidelines of 5% which may bring additional health benefits.^1 31^ This finding may suggest that reductions in energy intake from sugar were reducing in concert with overall energy intake and indeed may have been driving it. However, the magnitude of calories associated with the reduction in free sugars, compared with the counterfactual scenario in both adults and children, was modest and thus potentially too small to reflect significant changes in the percentage of energy from sugar. In children, a daily reduction of 4.8 g sugar equates to approximately 19.2 kilocalories out of an approximate daily intake of approximately 2000 kilocalories which is equivalent to approximately 1% reduction in energy intake. Furthermore, overall measures of dietary energy are also likely to involve a degree of error reducing the level of precision in any estimates.

Our estimates of changes in sugar consumption in relation to SDIL suggest that adults may have experienced a greater absolute reduction in sugar than children, which is not consistent with estimates of the distributional impact of the policy.^32^ However, our understanding may be aided by the visualisations afforded by graphical depictions of our ITS graphs. Children’s consumption of sugar at the beginning of the study period, particularly in soft drinks, was higher than in adults but reducing at a steeper trajectory, which will have influenced our estimated counterfactual scenario of what would have happened without the SDIL. This steep downward trajectory could not have continued indefinitely as there is a lower limit for sugar consumption. No account for this potential ‘floor effect’ was made in the counterfactual. Adults had a lower baseline of sugar consumption, but their trajectory of sugar consumption decreased at a gentler trajectory, potentially allowing more scope for improvement over the longer run.

Reductions in the levels of sugar in food and drink may have also impacted different age groups and children and adults differently. For example, the largest single contributor to free sugars in younger children aged 4–10 years is cereal and cereal products, followed by soft drinks and fruit juice. By the age of 11–18 years, soft drinks provide the largest single source (29%) of dietary free sugar. For adults the largest source of free sugars is sugar, preserves and confectionery, followed by non-alcoholic beverages.^5^

### Strengths and limitations

The main strengths of the study include the use of nationally representative data on individual consumption of food and drink in and out of the home using consistent food diary assessment over a 4-day period, setting it apart from other surveys which have used food frequency questionnaires, 24 hour recall, shortened dietary instruments or a mixture of these approaches across different survey years.^33^ The continual collection of data using consistent methods enabled us to analyse dietary sugar consumption and energy quarterly over 11 years (or 45 time points) including the announcement and implementation period of the SDIL. Information on participant age allowed us to examine changes in sugar consumption in adults and children separately. Limited sample sizes restricted our use of weekly or monthly data and prevented us from examining differences between sociodemographic groups. At each time point we used protein consumption in food and drink as a non-equivalent control category, strengthening our ability to adjust for time-varying confounders such as contemporaneous events. The trends in counterfactual scenarios of sugar consumption and energy from free sugar as part of total energy were based on trends from April 2008 to the announcement of the UK SDIL (March 2016); however, it is possible that the direction of sugar consumption may have changed course. Ascribing changes in free sugar consumption to the SDIL should include exploration of other possible interventions that might have led to a reduction in sugar across the population. We are only aware of the wider UK government’s voluntary sugar reduction programme implemented across overlapping timelines (2015–2020) and leading to reductions in sugar consumption that were well below the targets set.^30^ In turn, under-reporting of portion sizes and high energy foods, which may be increasingly seen as less socially acceptable, has been suggested as a common error in self-reported dietary intake with some groups including older teenagers and females, especially those who are living with obesity, more likely to underestimate energy intake.^34 35^ However, there is no evidence to suggest this would have changed as a direct result of the SDIL.^36^

## Conclusions

Our findings indicate that the UK SDIL led to reductions in consumption of dietary free sugars in adults and children 1 year after it came into force. Energy from free sugar as a proportion of overall energy intake was falling prior to the UK SDIL but did not change in relation to the SDIL, suggesting that a reduction in sugar may have driven a simultaneous reduction in overall energy intake.

## Data Availability

Data are available in a public, open access repository. Data from the National Diet and Nutrition Survey years 1–11 (2008–09 to 2018–19) can be accessed on the UK Data Service (https://ukdataservice.ac.uk/).
