# Supplementary material for: Estimated changes in free sugar consumption one year after the UK soft drinks industry levy came into force: controlled interrupted time series analysis of the National Diet and Nutrition Survey (2011–2019)
Source: J Epidemiol Community Health. 2024 Jul 9;78(9):578–84. doi: 10.1136/jech-2023-221051 (PMC11347969; doi:10.1136/jech-2023-221051)
Supplement: Supplementary data [file jech-2023-221051supp001.pdf]

Supplementary Material

Online supplemental text 1

Following the implementation of the UK SDIL soft drinks with less than 5 grams (g) sugar per 100ml were not liable for the levy, soft drinks with between 5g and 8g of sugar per 100ml were subject to a levy of £0.18 per litre and soft drinks with more than 8g sugar per 100ml subject to a higher levy of £0.24 per litre. Fruit juice, milk and milk-based drinks, powder used to make drinks and alcoholic drinks were exempt from the levy, regardless of their sugar content.

Online supplemental text 2

Participants report all food and drinks (including brand names, portion sizes and if home-cooked, ingredients and recipe) consumed inside and outside of their home over three or four days. Data were collected in-person by trained fieldworkers with parents/carers asked to help their children (12 years or less) to complete the food diary, or to complete it for them. Food and drink data from diaries was converted to nutrient intake data by linking recorded foods to the UK nutrient databank.

Online supplemental text 3

The term “free sugars” is defined by Scientific Advisory Committee on Nutrition (SACN) to include all monosaccharides and disaccharides added to foods and drinks by manufacturers, cooks, or consumers as well as those naturally present sugars in honey, syrups, unsweetened fruit juice, purees, pastes, and extruded products[5]. Naturally occurring sugars contained within intact plant cell walls (mostly fruits and vegetables, which can be fresh or processed) or in milk (lactose) are excluded from the definition of free sugar. This definition of free sugars is very similar to the published WHO definition of free sugars.

Online supplemental text 4

Protein intake was measured and matched to the outcomes of interest – i.e. absolute protein intake was the control for absolute free sugar intake and % energy from protein as the control for %energy from free sugars; protein intake from food and drink was the control for free sugar intake from food and drink.

Online supplemental text 5

The model specification is:

$Yt = \beta_0 + \beta_1 T + \beta_2 Z + \beta_3 Z{\cdot}T + \beta_4 A + \beta_5 At + \beta_6 Z{\cdot}A + \beta_7 Z{\cdot}At$

Yt	Mean sugar consumed in soft drinks per person/ day at quarter t (t=1,…,45)
T	Quarterly time point number from the start of the study (eg: 1:45)
Z	Control category (eg: protein consumption = 0, soft drinks = 1)
A	Pre-announcement = 0, post-announcement = 1
A_t	Quarterly time point number since announcement [0{33}, 1:12]

And coefficients are interpreted as follows:

β_0 : Intercept for protein consumption
β_1 : Trend change in consumption of protein per day (pre announcement)
β_2 : Intercept for sugar from soft drinks
β_3 : Trend change in consumption of sugar from soft drinks across time

β_4 : Step change in consumption of protein (at announcement)
β_5 : Trend change in consumption of protein per day (post announcement)
β_6 : Step change in consumption of sugar from soft drinks (at announcement)
β_7 : Trend change in consumption of sugar from soft drinks (post announcement)

Online supplemental text 6

Changes to Protocol: In the study protocol[26] we had planned to examine free sugar consumption changes across different categories of drink (depending on their sugar-content and if they were levy-eligible) and by socio-demographic characteristics at the individual or household-level (including age, gender, equivalised household income and educational qualifications and social class of the head of the household). However, the modest number of individuals represented at each time point (~ 125 adults and 125 children) limited the breakdown of our analysis into sub-groups beyond examination of adults and children separately and food and drinks were not broken down into individual food or drink-types (e.g.: juice only).

Supplementary Figure legends

Figure S1: Observed and modelled energy from free-sugar in food and drinks as a % of energy from food and drinks in adult/children from April 2008 to March 2019. Red points show observed data and solid red lines (with light red shadows) shows modelled data (and 95% confidence intervals) of energy from free-sugar in drinks as a % of total energy intake. The dashed red line indicates the counterfactual line based on preannouncement trends and had the announcement and implementation not happened. Modelled energy from protein consumption in food and drinks (control group) was removed from the graph to include resolution but is available in the supplementary section. The first and second dashed lines indicate the announcement and implementation of SDIL, respectively.

Figure S2: Observed and modelled energy from free-sugar in drinks as a % of energy from drinks in adult/children from April 2008 to March 2019. Red points show observed data and solid red lines (with light red shadows) shows modelled data (and 95% confidence intervals) of energy from free-sugar in drinks as a % of total energy intake. The dashed red line indicates the counterfactual line based on preannouncement trends and had the announcement and implementation not happened. Modelled energy from protein consumption in drinks (control group) was removed from the graph to include resolution but is available in the supplementary section. The first and second dashed lines indicate the announcement and implementation of SDIL, respectively.

Figure S3: Observed and modelled daily consumption (g) of protein from drink products per adult/children from April 2008 to March 2019. Red points and solid red lines (with 95% CI) show modelled protein consumption from drinks. The dashed red line indicates the counterfactual line based on preannouncement trends in protein consumption and had the announcement and implementation not happened. The light blue lines represents free-sugar consumption (g) from drinks as shown in figure 1. The first and second dashed lines indicate the announcement and implementation of SDIL, respectively.

Figure S4: Observed and modelled daily consumption (g) of protein from food and drink products per adult/children from April 2008 to March 2019. Red points and solid red lines (with 95% CI) show modelled protein consumption from food and drinks. The dashed red line indicates the counterfactual line based on preannouncement trends in protein consumption and had the announcement and implementation not happened. The light blue lines represent free-sugar consumption (g) from food and drinks as shown in figure 2. The first and second dashed lines indicate the announcement and implementation of SDIL, respectively.

Figure S5: Observed and modelled energy from protein as a % of energy from drinks in adult/children from April 2008 to March 2019. Red points show observed data and solid red lines (with light red shadows) shows modelled data (and 95% confidence intervals) of energy from protein in drinks as a % of total energy intake. The dashed red line indicates the counterfactual line based on preannouncement trends and had the announcement and

implementation not happened. The light blue lines represent energy from free-sugar from drinks, as shown in figure 4. The first and second dashed lines indicate the announcement and implementation of SDIL, respectively.

Figure S6: Observed and modelled energy from protein in food and drinks as a % of energy from food and drinks in adult/children from April 2008 to March 2019 April 2008 to March 2019. Red points show observed data and solid red lines (with light red shadows) shows modelled data (and 95% confidence intervals) of energy from protein in food and drinks as a % of total energy intake. The dashed red line indicates the counterfactual line based on preannouncement trends and had the announcement and implementation not happened. The light blue lines represent energy from free-sugar from food and drinks, as shown in figure 3. The first and second dashed lines indicate the announcement and implementation of SDIL, respectively.

S1

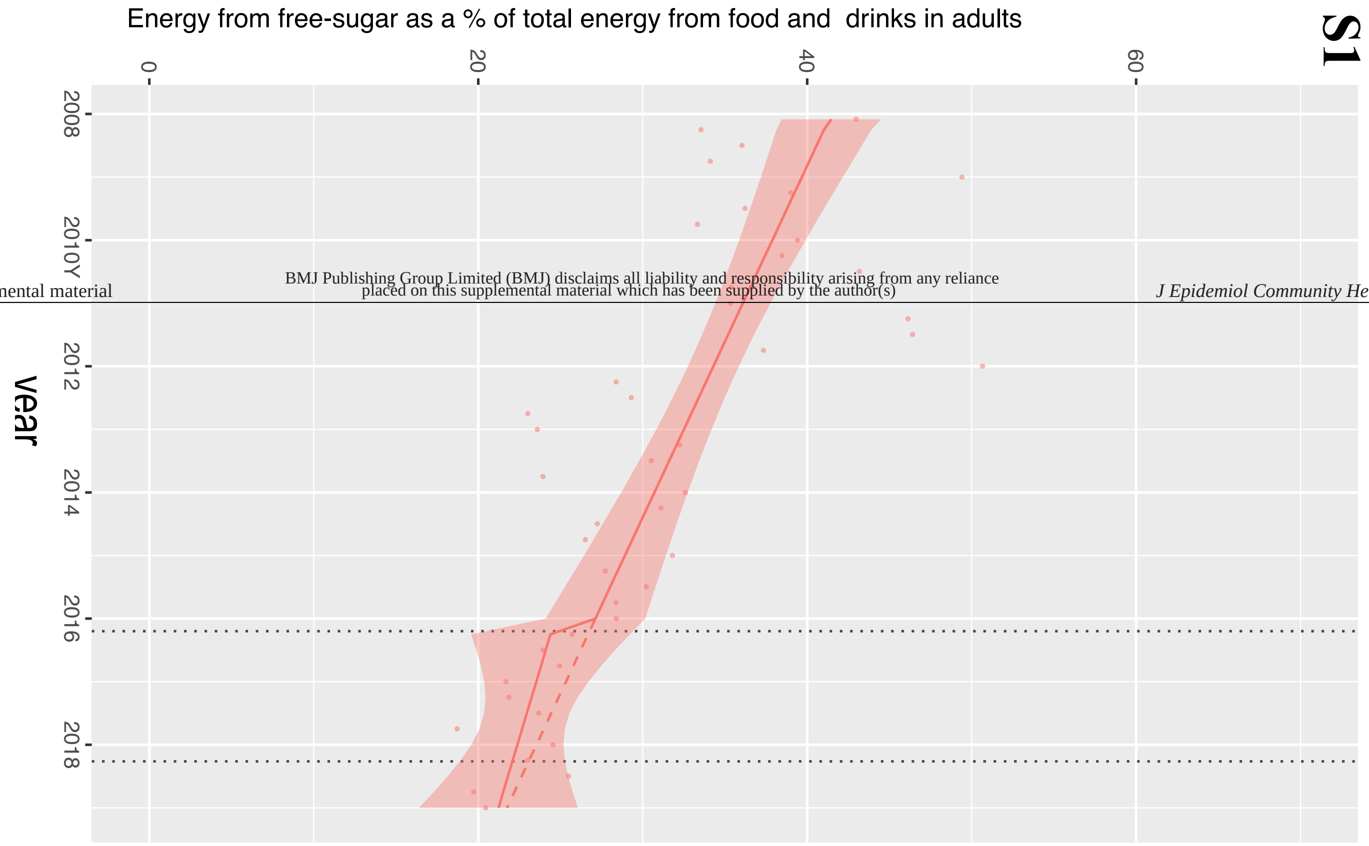
Energy from free-sugar as a % of total energy from food and drinks in adults
year
0
20
40
60
2008
2010
2012
2014
2016
2018

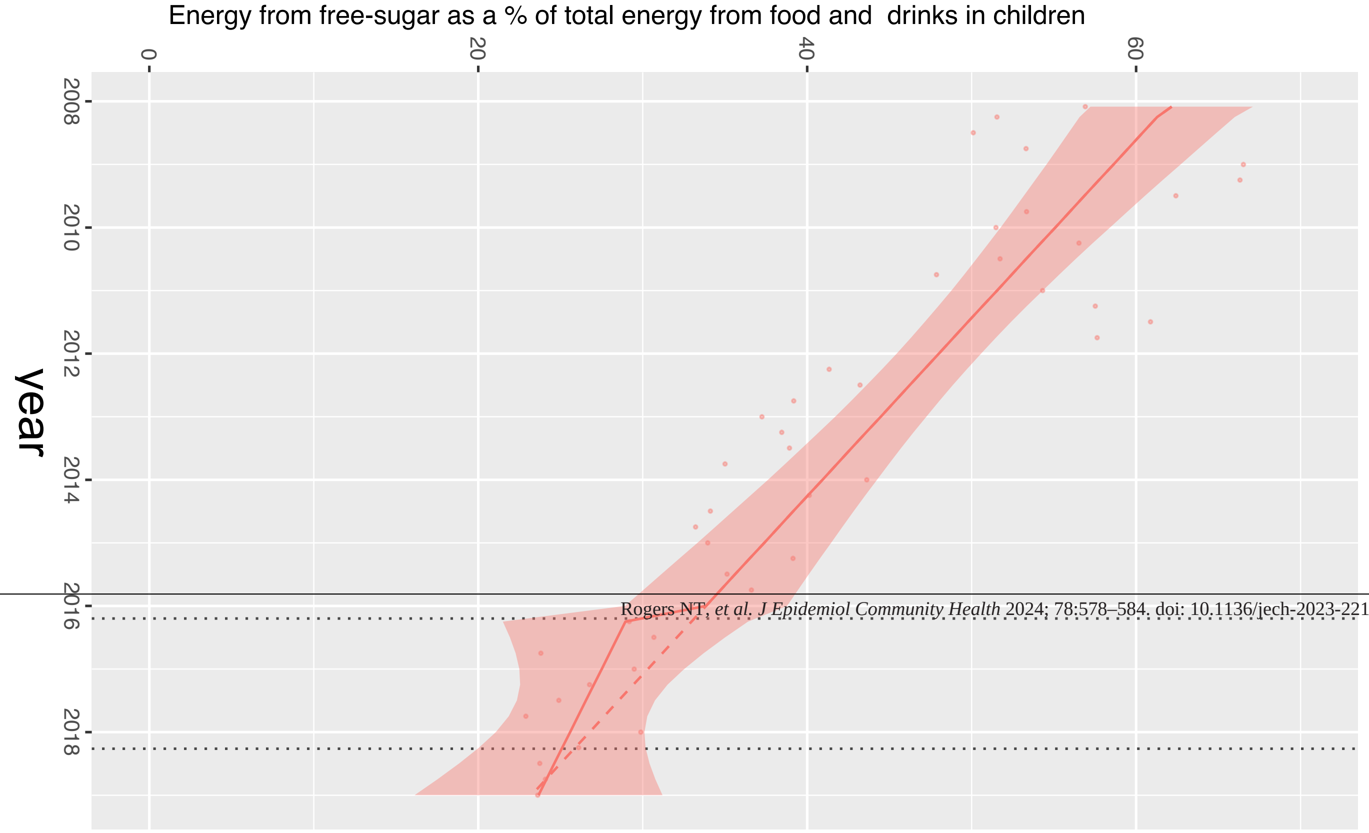
Energy from free-sugar as a % of total energy from food and drinks in children
year
0
20
40
60
2008
2010
2012
2014
2016
2018

S2

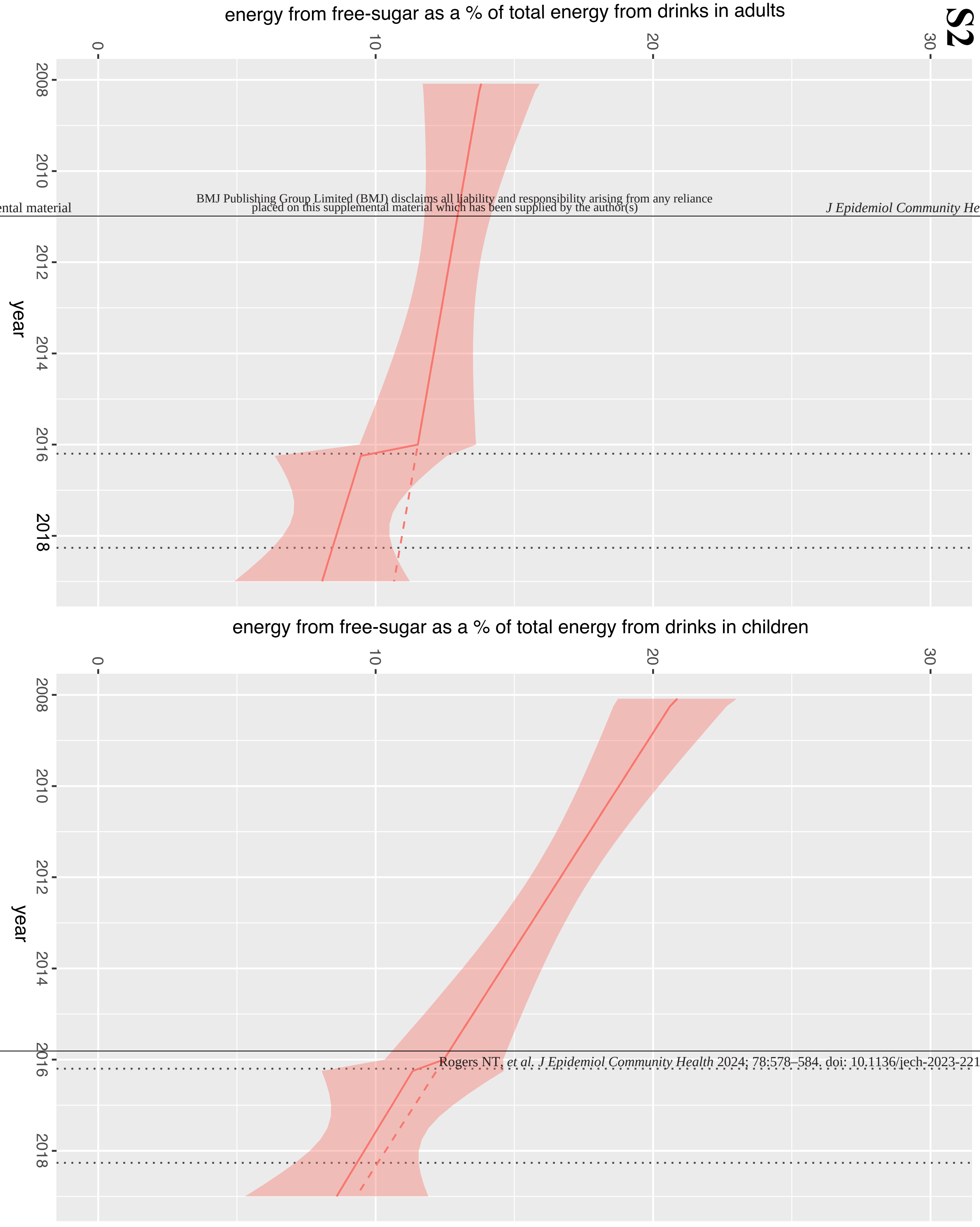
energy from free-sugar as a % of total energy from drinks in adults
0
10
20
30
year
2008
2010
2012
2014
2016
2018
energy from free-sugar as a % of total energy from drinks in children
0
10
20
30
year
2008
2010
2012
2014
2016
2018

S3

sugar and protein (g) consumption per day from drinks in adults

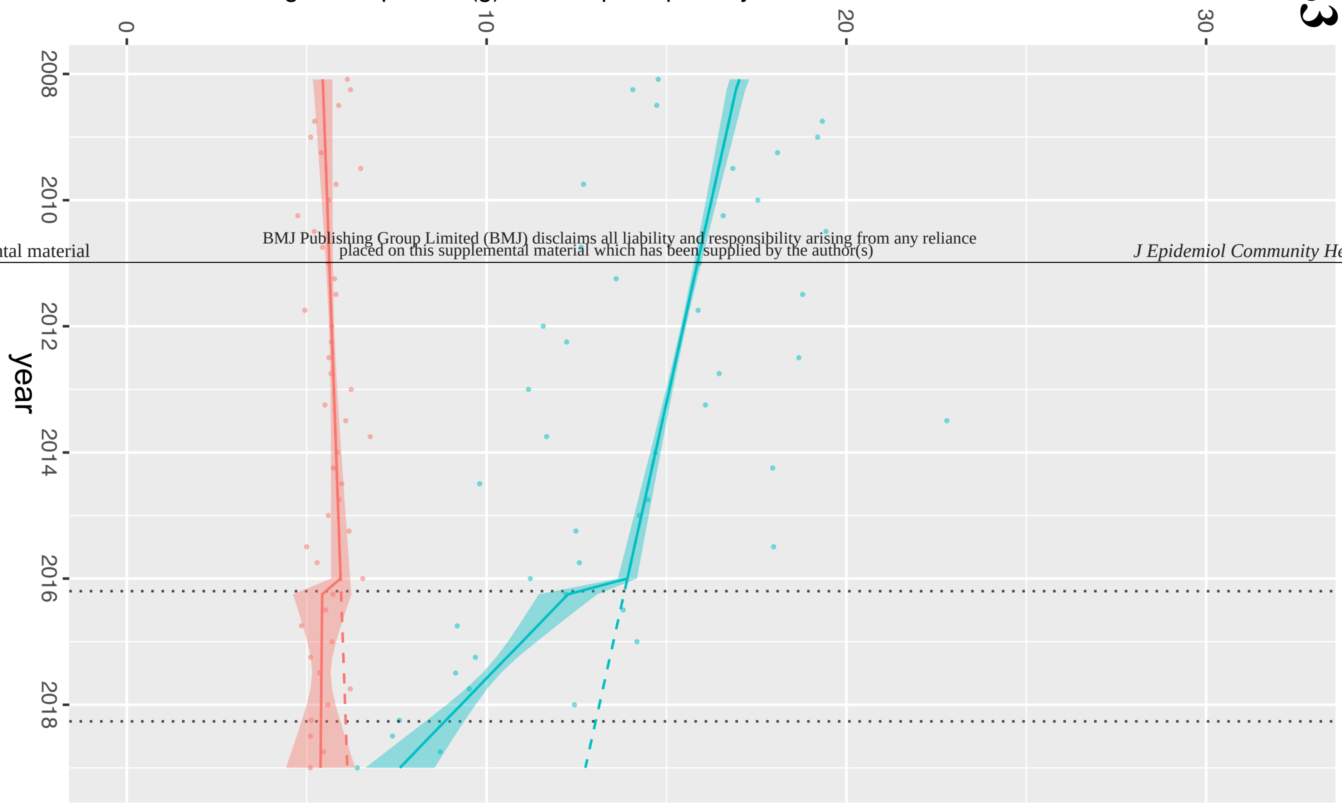

sugar and protein (g) consumption per day from drinks in children

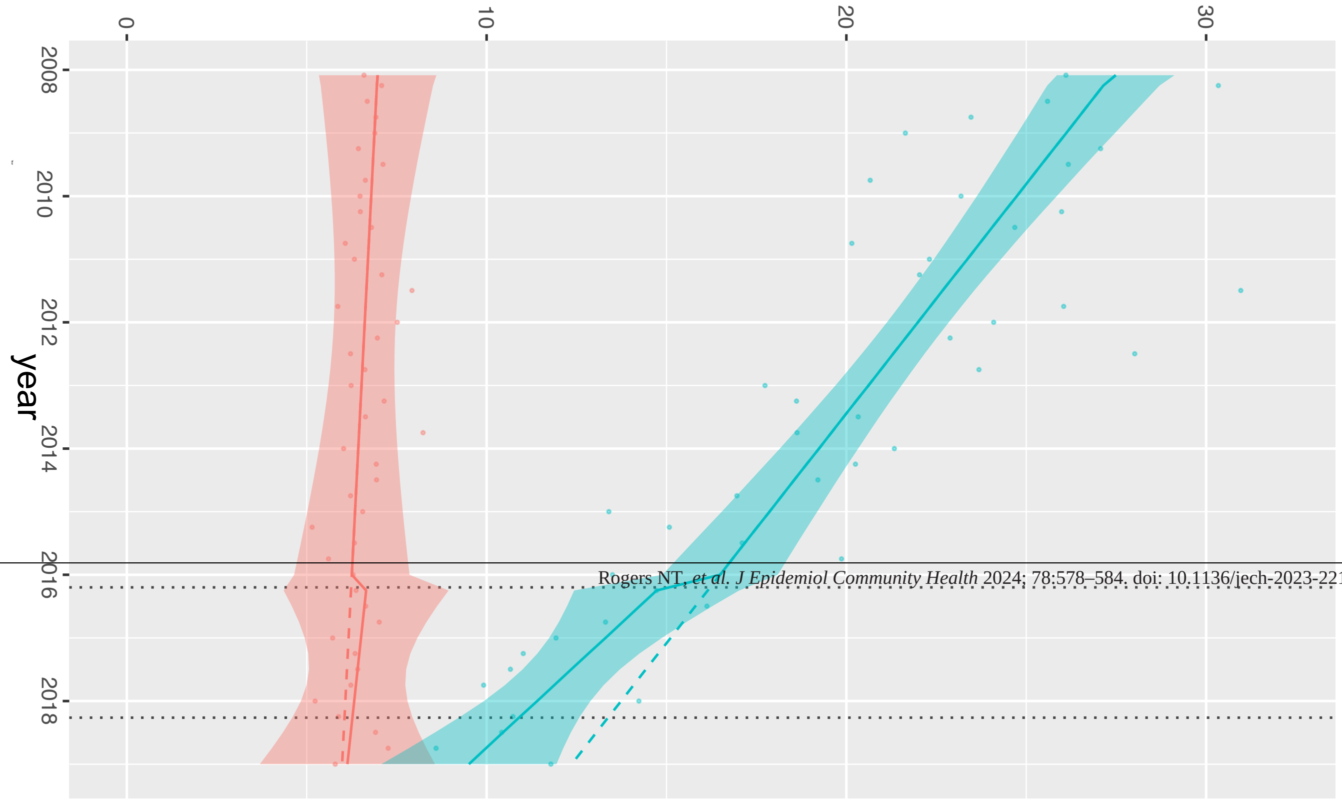

S4

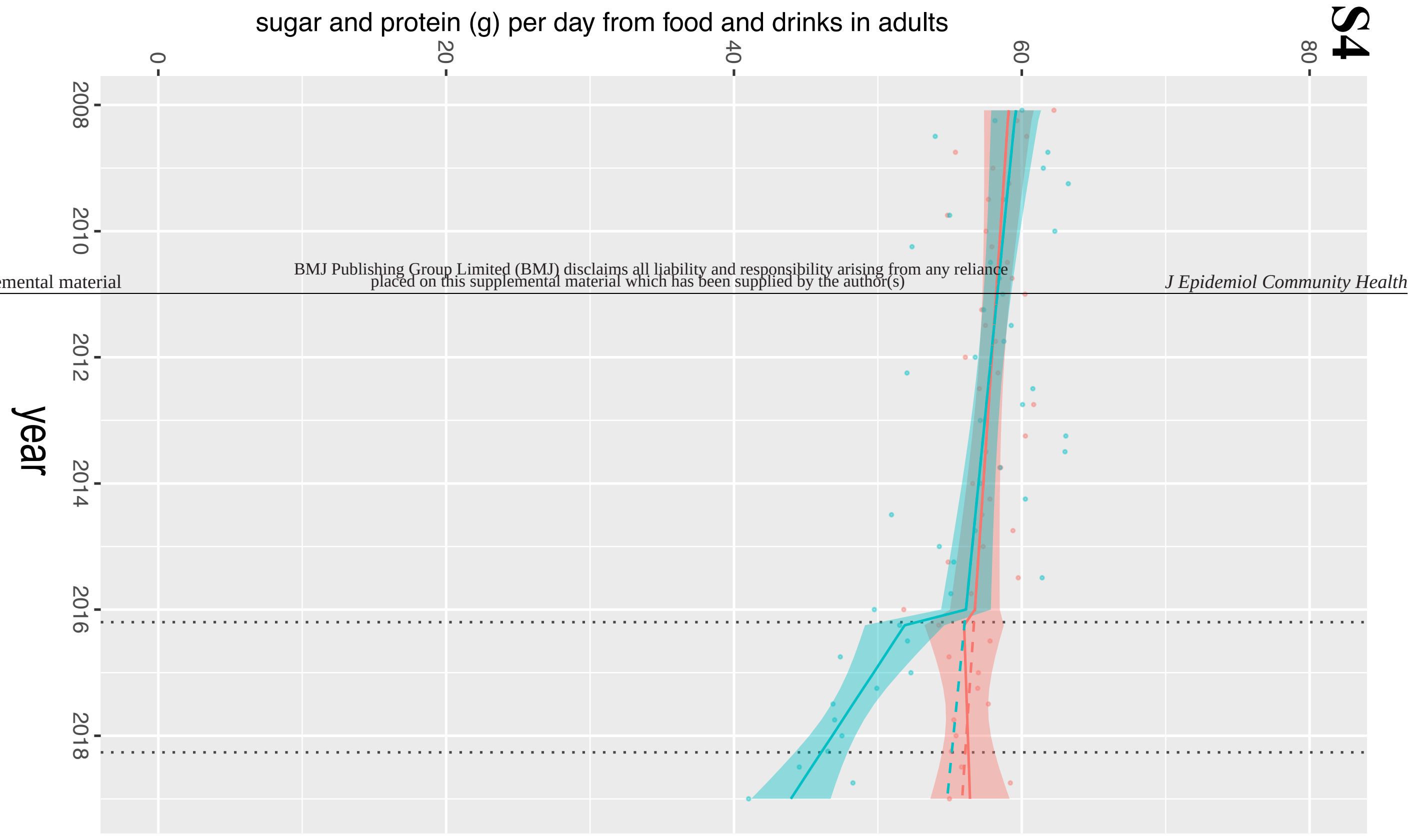
sugar and protein (g) per day from food and drinks in adults
year
0
20
40
60
80
2008
2010
2012
2014
2016
2018

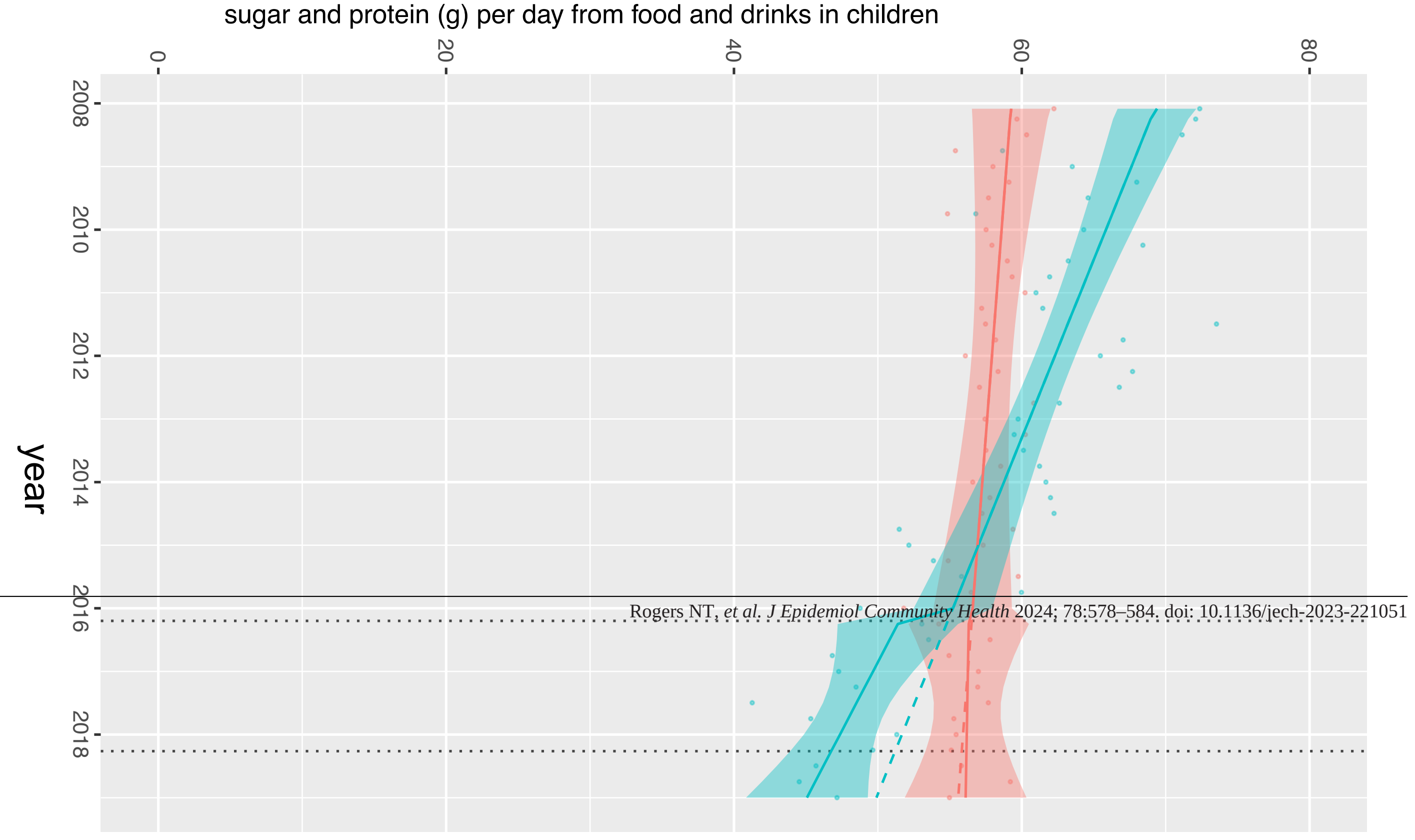
sugar and protein (g) per day from food and drinks in children
year
0
20
40
60
80
2008
2010
2012
2014
2016
2018

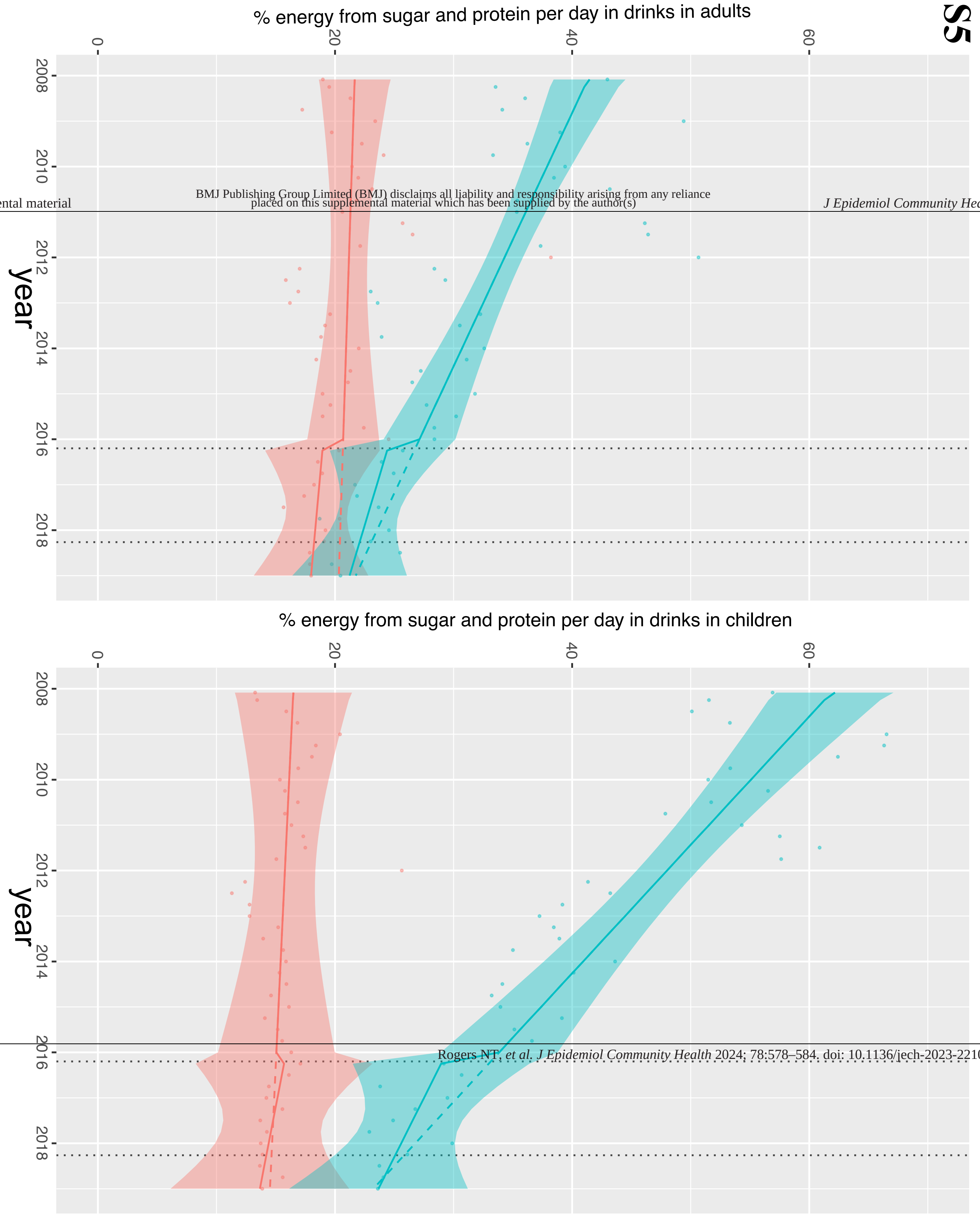
S5
% energy from sugar and protein per day in drinks in adults
% energy from sugar and protein per day in drinks in children
year
0
20
40
60
2008
2010
2012
2014
2016
2018

S6

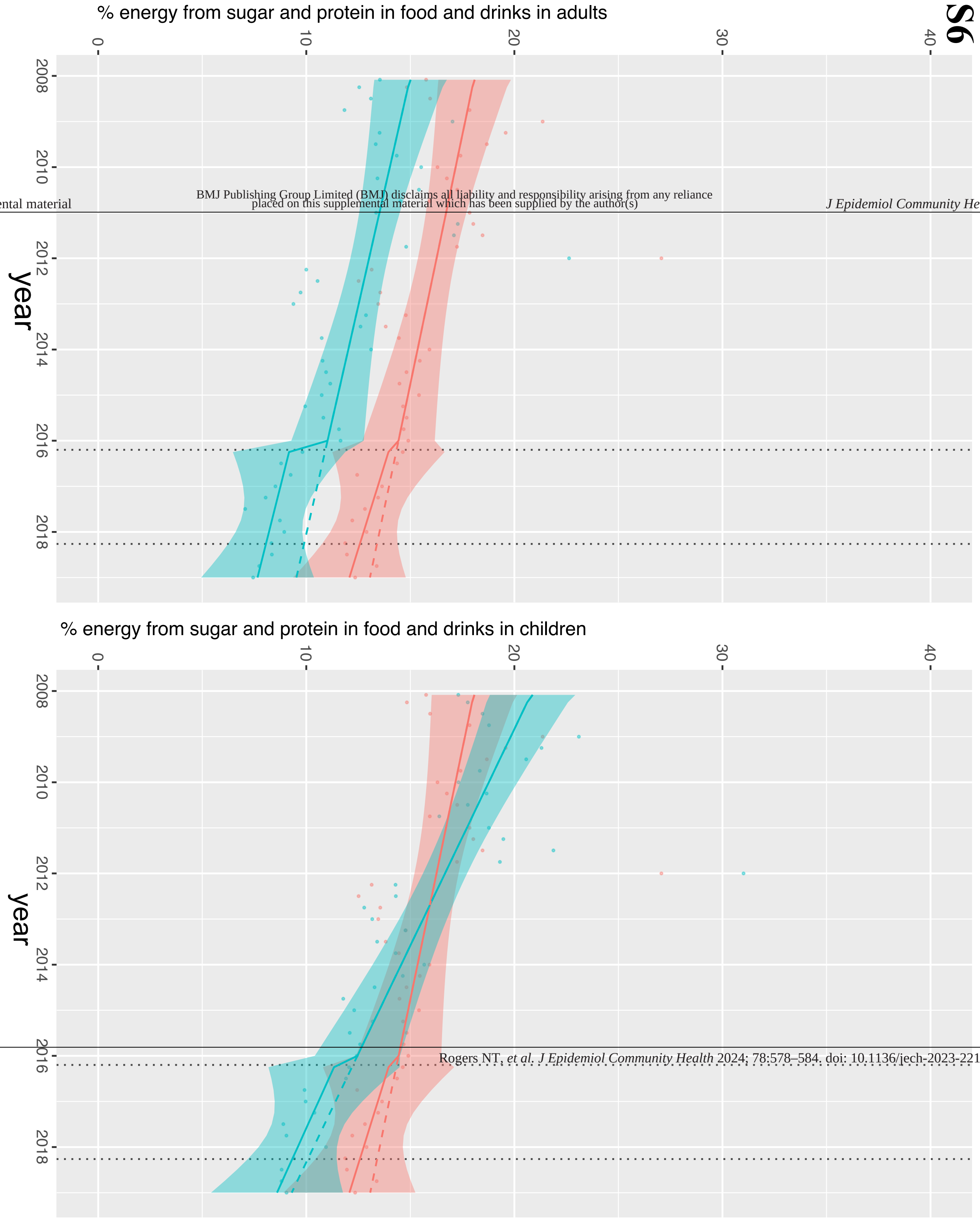
% energy from sugar and protein in food and drinks in adults
0
10
20
30
40
2008
2010
2012
2014
2016
2018
year
% energy from sugar and protein in food and drinks in children
0
10
20
30
40
2008
2010
2012
2014
2016
2018
year
